# Epidemiological Trends and Clinicopathological Characteristics of Oral Leukoplakia: A Retrospective Analysis From a Single Institution in Chennai, Tamil Nadu, India

**DOI:** 10.7759/cureus.61590

**Published:** 2024-06-03

**Authors:** Deeksheetha Prabhu Venkatesh, Karthikeyan Ramalingam, Pratibha Ramani, Murugesan Krishnan, Jayanth Kumar Vadivel

**Affiliations:** 1 Oral Pathology and Microbiology, Saveetha Dental College and Hospitals, Saveetha Institute of Medical and Technical Sciences, Saveetha University, Chennai, IND; 2 Oral and Maxillofacial Surgery, Saveetha Dental College and Hospitals, Saveetha Institute of Medical and Technical Sciences, Saveetha University, Chennai, IND; 3 Oral Medicine and Radiology, Saveetha Dental College and Hospitals, Saveetha Institute of Medical and Technical Sciences, Saveetha University, Chennai, IND

**Keywords:** prevalence studies, surgical excision, pharmacotherapy, tobacco-related oral lesions, proliferative verrucous leukoplakia, oral submucous fibrosis, epithelial dysplasia, oral mucosal lesions, oral potentially malignant disorders, oral leukoplakia

## Abstract

Background

India has a high prevalence of oral potentially malignant disorders and malignant transformation. Cases of oral leukoplakia are not commonly encountered, and only a small cohort of patients undergo biopsies for the same. This study aims to assess the various etiological factors causing leukoplakia, the clinical features, histopathological findings, and treatment received by the patients who were histopathologically diagnosed with oral leukoplakia.

Methodology

Oral leukoplakia cases were included in this study from total biopsy samples received in the oral pathology department. Details were collected from the Dental Information Archival Software of our institution. The period analyzed was from January 1, 2021, to December 31, 2023. Relevant clinical and histopathological details were retrieved and tabulated. Statistical analysis (chi-square test) was used to assess the association between the clinicopathological parameters using SPSS software version 21.0 (IBM Corp., Armonk, NY, USA) with a significance level set at a p-value <0.05.

Results

A total of 76 oral leukoplakia cases were retrieved from 2,600 biopsy samples. The prevalence of oral leukoplakia was 3.1% to 3.4% for the three years. Leukoplakia was commonly observed in those aged 51 to 60 years (33%). Overall, 21% of the patients with leukoplakia showed severe epithelial dysplasia, 22% showed mild epithelial dysplasia, and 39% showed moderate epithelial dysplasia. Moreover, 30% of the patients presented with leukoplakia and oral submucous fibrosis and showed varying degrees of epithelial dysplasia. Finally, 45% of the patients were managed conservatively using pharmacotherapy.

Conclusions

Severe epithelial dysplasia was commonly associated with oral leukoplakia. Oral submucous fibrosis was also found to be associated with leukoplakia and showed epithelial dysplasia. None of our proliferative verrucous leukoplakia cases showed any association with oral submucous fibrosis. Surgical management was the preferred treatment.

## Introduction

Oral potentially malignant disorders (OPMDs) occur at high frequencies in the Indian population. The prevalence of OPMDs in India is estimated to be 13.2% to 13.9% [1]. Oral leukoplakia is one of the most common OPMDs according to the World Health Organization (WHO). WHO defines it as a non-scrapable white patch or plaque that cannot otherwise be characterized clinically or pathologically as any other disease. The prevalence of oral leukoplakia is estimated at 1.0% to 4.2% worldwide [2]. Dentists commonly encounter oral leukoplakia in their daily practice, and a definitive diagnosis of oral leukoplakia is made by histopathological examination post-biopsy. Despite its benign nature, leukoplakia poses a diagnostic challenge due to its diverse etiology, variable clinical presentations, and potential for malignant transformation [3]. The clinical significance of leukoplakia is significant due to its association with potentially malignant disorders [4]. Hence, its management requires a nuanced approach informed by understanding its underlying pathophysiology and associated risk factors.

Clinically, leukoplakic lesions encompass the spectrum of morphological features ranging from homogenous white patches to verrucous or erythematous variants. Histopathological evaluation is a cornerstone that provides valuable insights into tissue architecture, cellular atypia, and other dysplastic changes within leukoplakia [5]. The pathogenesis of leukoplakia remains an enigma. Previous studies have proposed that the pathogenesis of leukoplakia is driven by somatic genetic mutations that affect the growth, survival, and cycle control of keratinocytes, reflecting as hyperkeratosis, hyperplasia, and dysplasia histopathologically, which translates clinically as changes in color and texture of the mucosa [6]. Genetic alterations in various oncogene drivers such as *EGFR*, *MYC*, *CCND1*, and *CDKN2A* have been found. Other genetic mutations such as DNA ploidy, loss of heterogeneity, and positive telomerase activity affecting predominantly *TP53* have also been identified. Other genes commonly associated are *FAT1*, *NOTCH1*, *KM12C*, and *PIK3CA* [7].

Although leukoplakia is considered a premalignant lesion, it is being considered under a broader term for tobacco usage in the form of smoking and smokeless tobacco. Leukoplakia poses a high chance of malignant transformation if the risk factors are not eliminated [8]. The risk factors for leukoplakia range from tobacco and alcohol usage to chronic irritation. It has a multitude of etiological factors that are implicated in its pathogenesis. Hence, timely diagnosis and appropriate treatment while removing the causative factors can prevent the malignant transformation of the disease. Based on previous studies, the management of leukoplakia has been an evolving landscape, such as conservative monitoring, pharmacotherapy, and surgical excision, while incorporating the emerging newer treatment modalities such as photodynamic therapy and topical chemopreventive agents [9].

As guardians of oral and systemic health, dentists and doctors play a pivotal role in the early detection and management of leukoplakia. They advocate for patient education, tobacco cessation, and regular surveillance. Hence, dental and medical professionals must understand the etiology, clinical manifestations, diagnostic approaches, and management strategies for leukoplakia. This study aims to assess the various etiological factors causing leukoplakia, the general clinical distribution, histopathological features, the risk of malignant transformation, and the treatment received by the patients.

## Materials and methods

In this hospital-based, retrospective study, data of patients who had undergone a biopsy between January 1, 2021, and December 31, 2023, at Saveetha Dental College and Hospitals, Chennai, India. All relevant data were obtained from the Dental Information Archival Software (DIAS). The Institutional Human Ethical Committee approved this study (bonafide letter number: IHEC/SDC/PhD/OPATH-1954/19/TH-001). All patients included in this study were clinically diagnosed with leukoplakia or proliferative verrucous leukoplakia, with or without oral submucous fibrosis.

Comprehensive demographic details were collected, including age, gender, and socioeconomic status. The chief complaints of the patients, such as the duration and nature of symptoms, were meticulously recorded. Clinical features, such as the size, location, and appearance of the lesions, were documented in detail. Clinical pictures of the patients were also obtained from the DIAS of our institution. These images provided visual documentation of the lesions, complementing the clinical descriptions. The incisional biopsy report of the suspected lesion sent to the Department of Oral and Maxillofacial Pathology for which histopathological diagnosis was conducted was also retrieved from the DIAS. The above-mentioned data were only collected after obtaining informed consent from the patients, and the study was conducted per the Declaration of Helsinki.

Histopathologically confirmed cases of leukoplakia were obtained by cross-referencing the digital cases database in the DIAS with the manual records to ensure completeness and accuracy of the collected data. For obtaining histopathological records, specific search terms such as “Hyperkeratosis” and “Leukoplakia” were used. Following the retrieval of the case data, leukoplakia cases were isolated for further analysis. To improve data integrity, digital records were cross-checked with manual registers to ensure no biopsy records were overlooked, thus guaranteeing the dataset’s reliability for future analysis and interpretation. This comprehensive approach ensured that the dataset was robust and reliable for subsequent analysis and interpretation, laying a solid foundation for the study’s findings.

The data obtained from the clinical and histopathological findings were meticulously organized and compiled in Microsoft Excel (Microsoft Corp., Redmond, WA, USA). This dataset included detailed information such as the patient’s age and gender, clinical and personal history, the site and clinical appearance of the lesion, and any history of deleterious habits such as tobacco and alcohol use. Each patient’s clinical presentation was thoroughly documented to ensure comprehensive data collection. The histopathological features were carefully noted and included the presence or absence of dysplasia. When dysplasia was present, it was categorized into mild, moderate, or severe epithelial dysplasia. Additionally, the presence or absence of oral submucous fibrosis was recorded based on histopathological examination. This granular level of detail was crucial for understanding the severity and nature of each patient’s condition. Information about the treatments administered to each patient was also compiled. This included pharmacotherapy, surgical excision, or physiotherapy, as applicable. Furthermore, the timeline and outcomes of postoperative review appointments were assessed to evaluate the effectiveness of the treatments and monitor patient recovery. Statistical analysis was performed using SPSS software version 20.0 (IBM Corp., Armonk, NY, USA). Initially, tests of normality were performed, and the Shapiro-Wilk test indicated that the data were non-parametric. Hence, the chi-square test was employed to compare variables, and any p-value of less than 0.05 was considered statistically significant.

## Results

**Figure 1:**
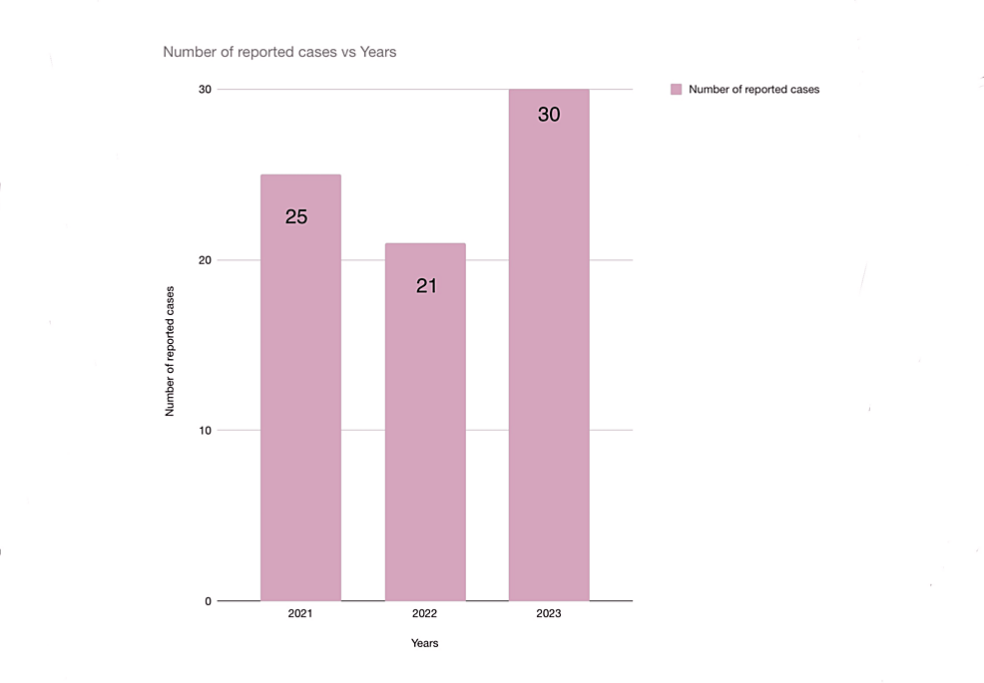
Graphical representation of the number of reported cases of leukoplakia in three years.

DIAS was used to retrieve relevant patient data and 2,600 records were identified. In total, 76 (2.92%) cases histopathologically diagnosed with oral leukoplakia were included for further evaluation. The year-wise distribution of oral leukoplakia cases was 25 cases in 2021, 21 in 2022, and 30 in 2023 (Figure 1).

**Figure 2:**
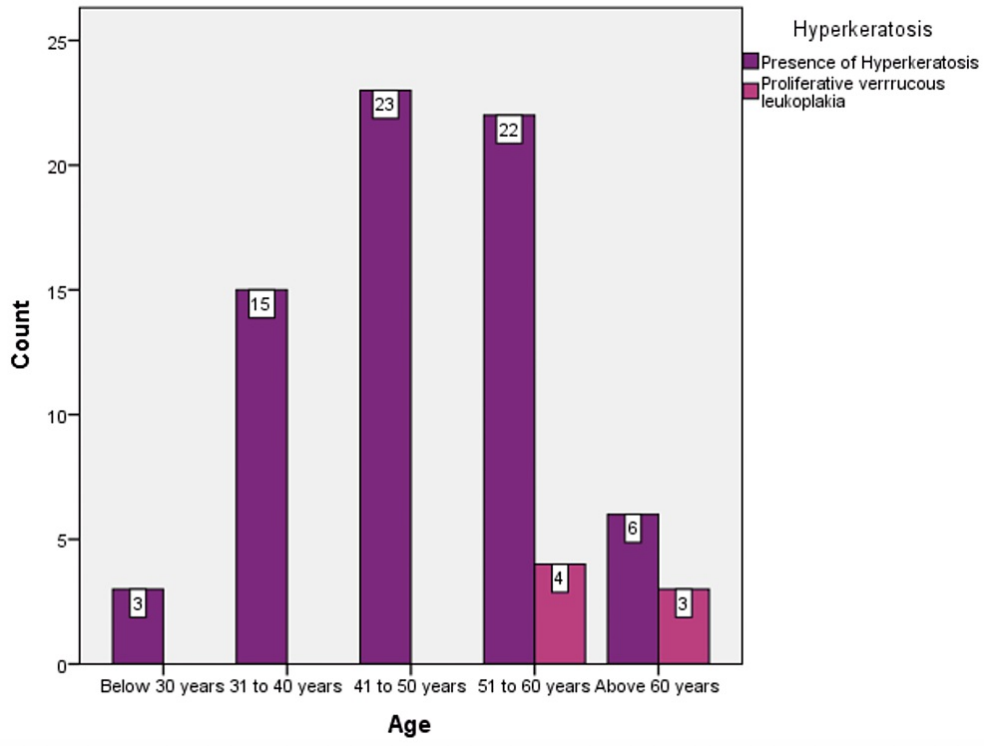
Distribution of observed lesions among different age groups.

The prevalence ranged between 3.1% to 3.4% in the three years. The age distribution of the patients included in the study was grouped as below 30 years (n = 3, 3.9%), 31 to 40 years (n = 15, 19.5%), 41 to 50 years (n = 23, 29.9%), 51 to 60 years (n = 26, 33.8%), and above 60 years (n = 9, 11.7%). There was a statistically significant difference in the incidence of leukoplakia and proliferative verrucous leukoplakia among the different age groups. (p = 0.021) (Figure 2).

**Figure 3:**
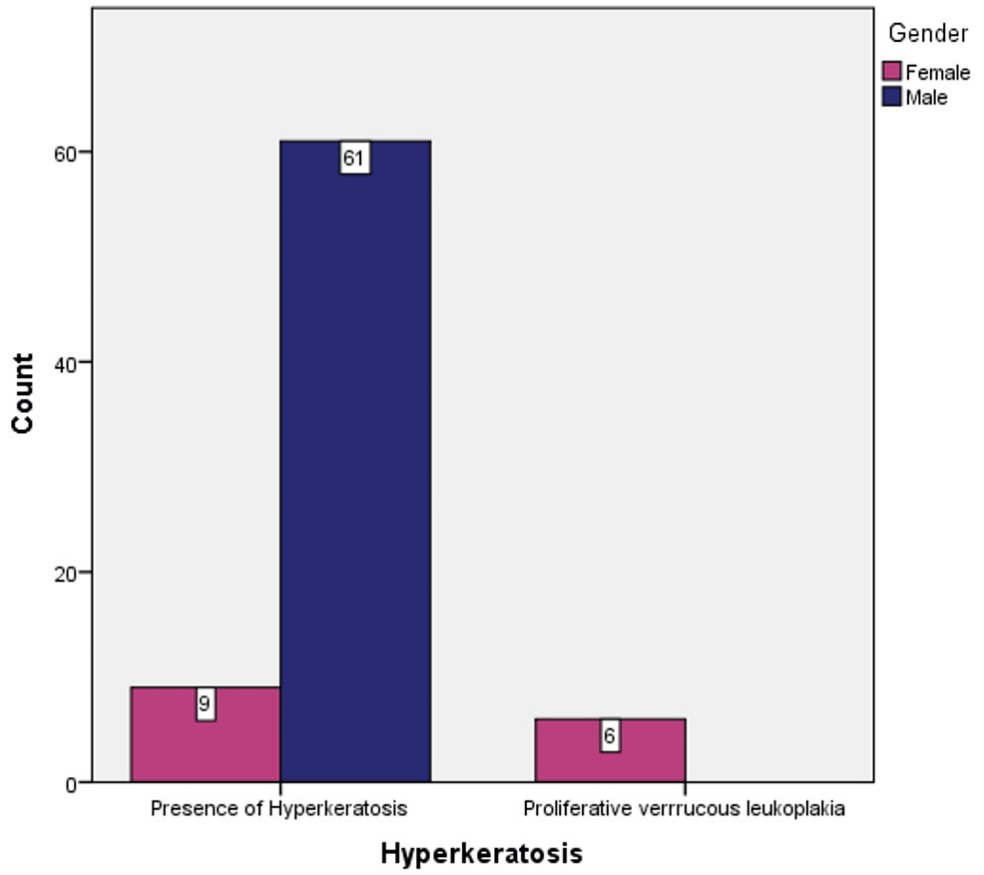
Gender comparison of hyperkeratosis and proliferative verrucous leukoplakia.

Only 15 (19.5%) patients were females, and 61 (79.2%) patients presenting with oral leukoplakia were males. Proliferative verrucous leukoplakia was only observed in female patients (p = 0.003) (Figure 3).

**Figure 4:**
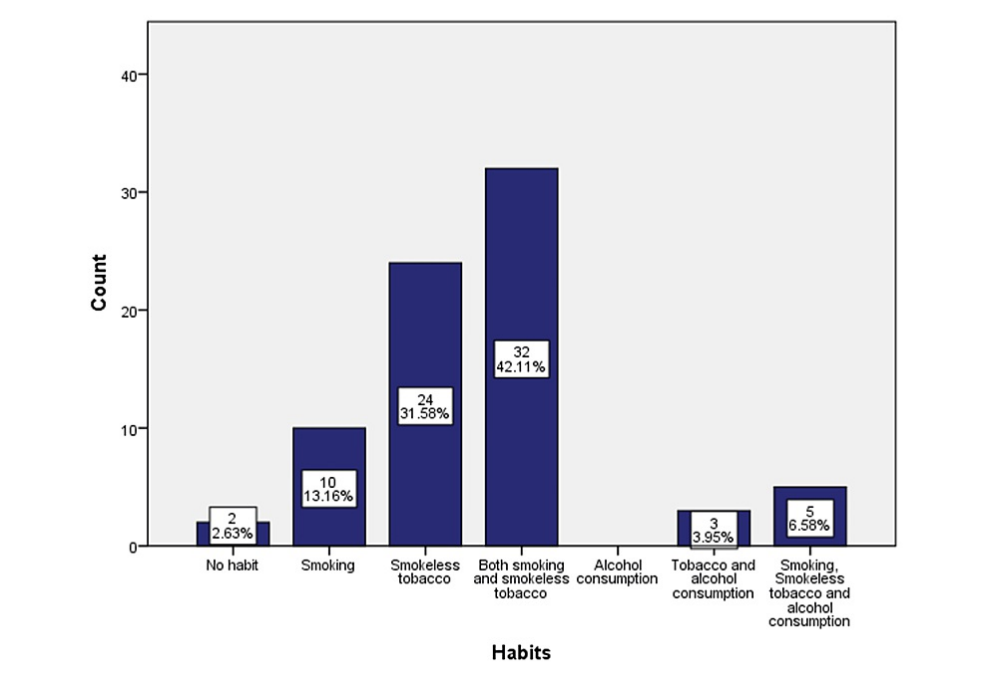
Graphical representation of deleterious habits observed in our study.

Smoking habits were noted in 10 (13.16%) patients, while 24 (31.6%) patients had the habit of using smokeless tobacco either in the form of betel nut, paan, or gutka. Moreover, 32 (42%) patients consumed tobacco both in the form of smoking and smokeless tobacco. Further, three (3.9%) patients had a smoking tobacco habit along with alcohol consumption. All three forms of adverse habits were noted in five (6.5%) patients. Only two (2.63%) patients did not present with any adverse habits. There were no patients who consumed only alcohol (Figure 4).

**Figure 5:**
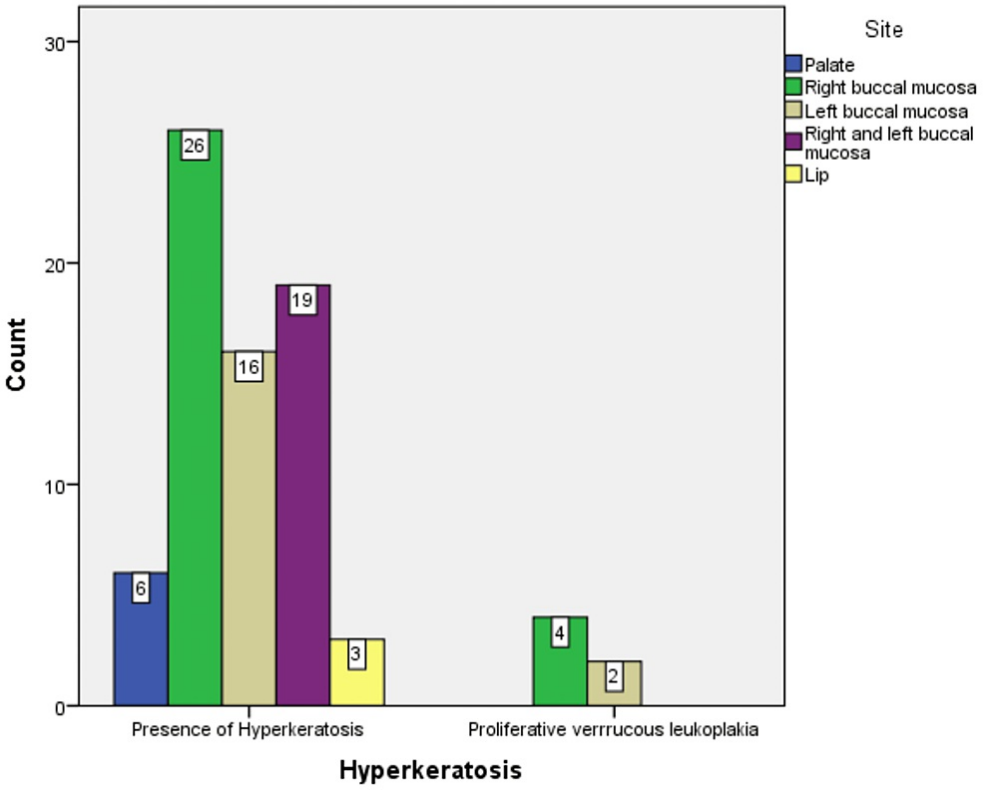
Site distribution of the observed cases.

Right buccal mucosa was the most common site noted in 30 (39%) patients, followed by bilateral involvement of the buccal mucosa noted in 19 (24%) patients. Overall, 16 (21%) patients showed involvement of the left buccal mucosa, six (8%) showed palate involvement, and three (4%) showed lip involvement (Figure 5).

**Figure 6:**
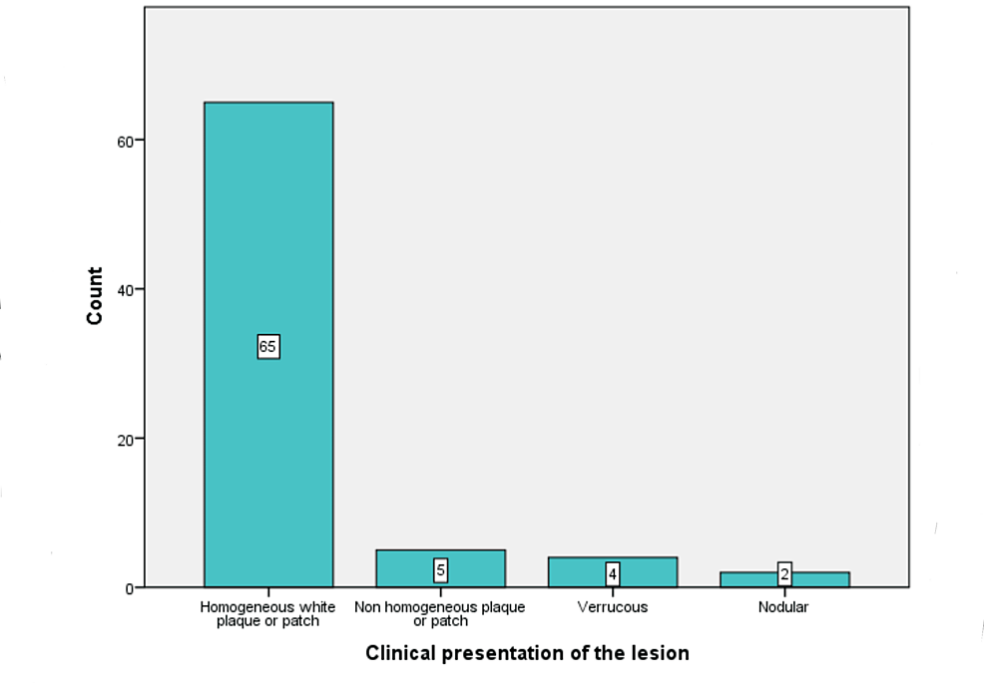
Clinical presentation of the observed lesions.

A homogeneous non-scrapable white plaque or patch was seen in 65 (84%) patients, five (6.5%) patients had non-homogeneous speckled plaque or patch, verrucous growths were noted in four (5.2%) patients, and nodular lesions were noted in two (2.6%) patients (Figure 6).

**Figure 7:**
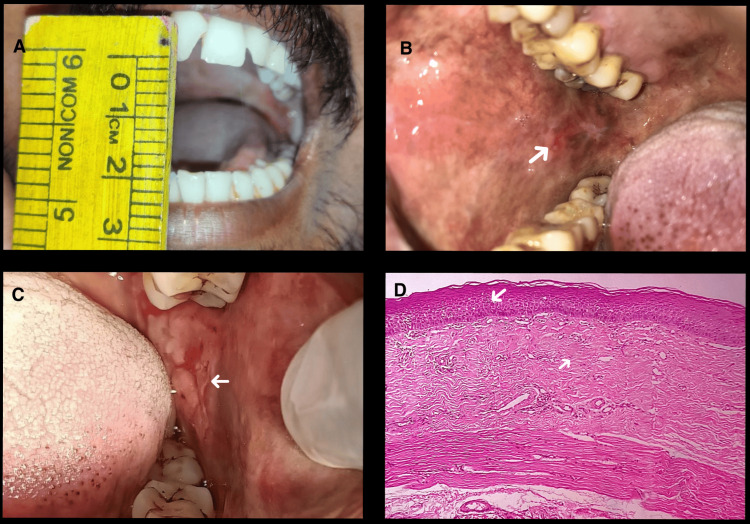
Clinical and histopathological images of leukoplakia with oral submucous fibrosis. A: Clinical image representing a restricted mouth opening. B: Discolored patch on the right buccal mucosa. C: Reddish-white plaque on the left buccal mucosa. D: Photomicrograph showing epithelial dysplasia with fibrosed connective tissue stroma (hematoxylin and eosin, 20×).

In 70 patients with hyperkeratosis, 23 (32.9%) also presented with oral submucous fibrosis (Figure 7).

**Figure 8:**
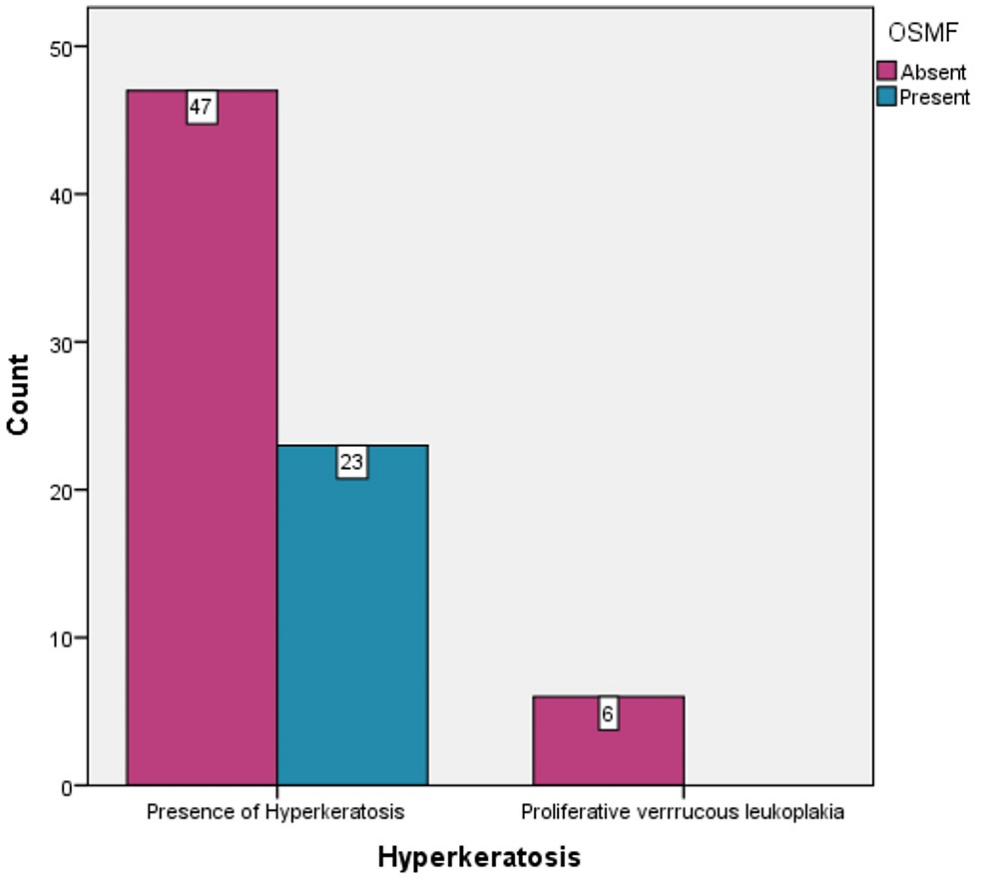
Distribution of observed cases with oral submucous fibrosis.

In all six patients with proliferative verrucous leukoplakia, there was no association with oral submucous fibrosis (p = 0.416) (Figure 8).

**Figure 9:**
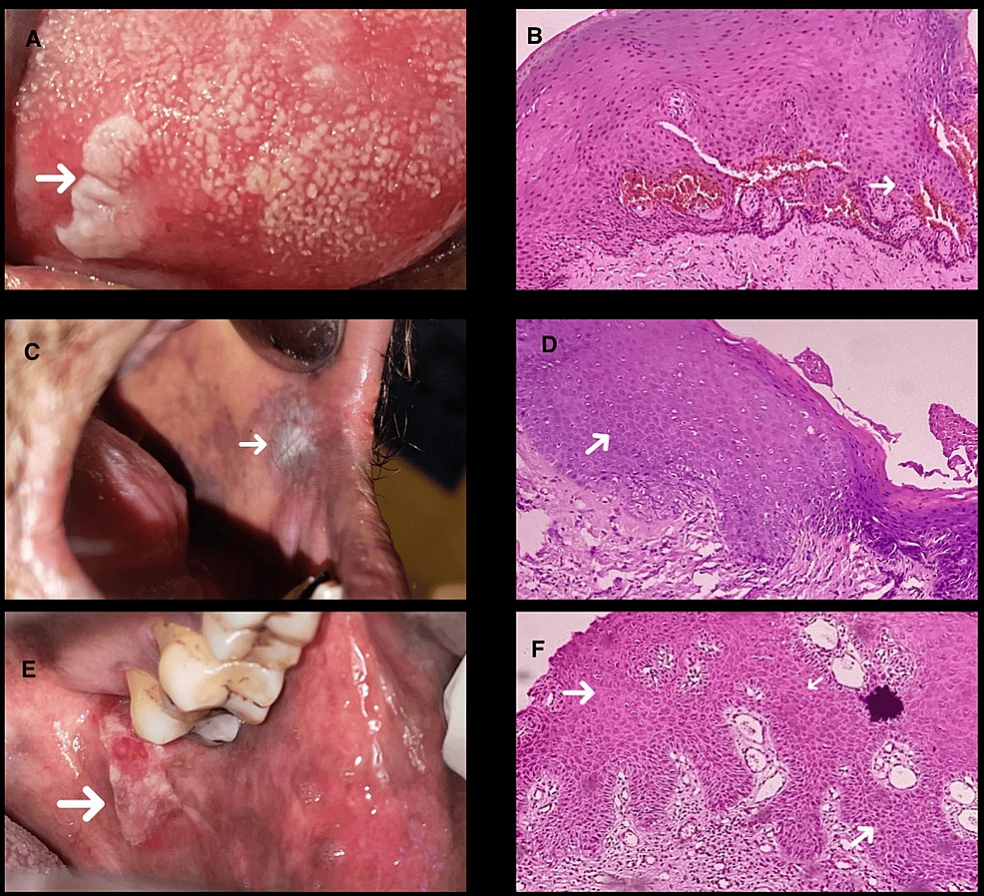
Clinical images of observed leukoplakia cases along with its histopathology. A: Clinical image of leukoplakia on the tongue. B: Photomicrograph showing mild epithelial dysplasia (hematoxylin and eosin, 10×). C: Clinical image of leukoplakia on the left buccal mucosa. D: Photomicrograph showing moderate epithelial dysplasia (hematoxylin and eosin, 10×). E: Clinical image of leukoplakia with ulcerated areas on the left buccal mucosa. F: Photomicrograph showing severe epithelial dysplasia (hematoxylin and eosin, 10×).

Of all the included subjects in the present study, eight (10%) had no evidence of epithelial dysplasia, 17 (22%) showed mild epithelial dysplasia, 30 (39%) showed moderate epithelial dysplasia, and 16 (21%) showed severe epithelial dysplasia (Figure 9).

**Figure 10:**
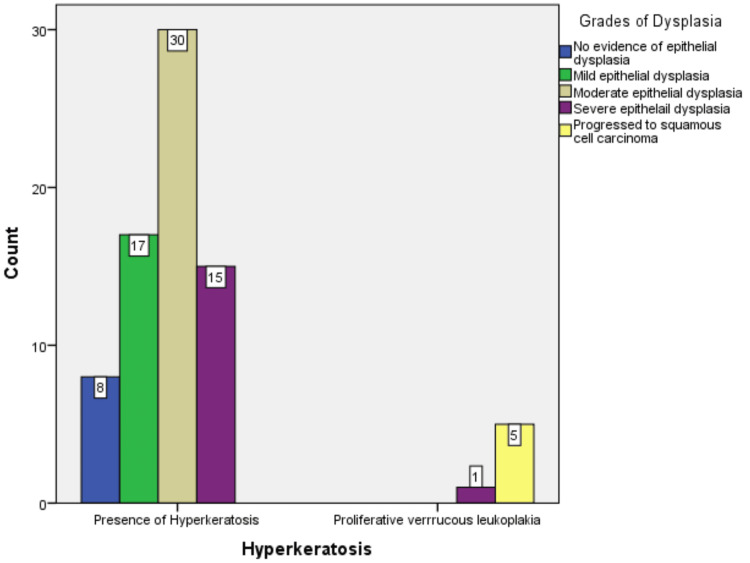
Grades of dysplasia among the observed lesions.

Five (7%) cases of leukoplakia progressed into squamous cell carcinoma. It was observed that all six cases of proliferative verrucous leukoplakia had progressed to squamous cell carcinoma (p < 0.001) (Figure 10).

In our study, 12 (16%) patients were afflicted with hypertension, while eight (10%) patients presented with diabetes mellitus. Six (8%) patients had both hypertension and diabetes. Four (33%) of the patients with hypertension had hyperkeratosis with moderate epithelial dysplasia and eight (66%) of the patients with hypertension had hyperkeratosis with severe epithelial dysplasia. Additionally, four (50%) of the patients with diabetes mellitus had hyperkeratosis with mild epithelial dysplasia, two (25%) patients only had hyperkeratosis, and two (25%) patients had hyperkeratosis with severe epithelial dysplasia. In patients with both diabetes and hypertension, four (66%) had proliferative verrucous leukoplakia, one (16%) had hyperkeratosis with moderate epithelial dysplasia, and one (16%) had hyperkeratosis with severe epithelial dysplasia. Ki-67 was assessed in only two patients with proliferative verrucous leukoplakia. The mean Ki-67 proliferation rate was 28%. All cases of proliferative verrucous leukoplakia showed malignant transformation to oral squamous cell carcinoma. Human papillomavirus and human immunodeficiency virus examinations were not performed on the patients included in our data set.

**Figure 11:**
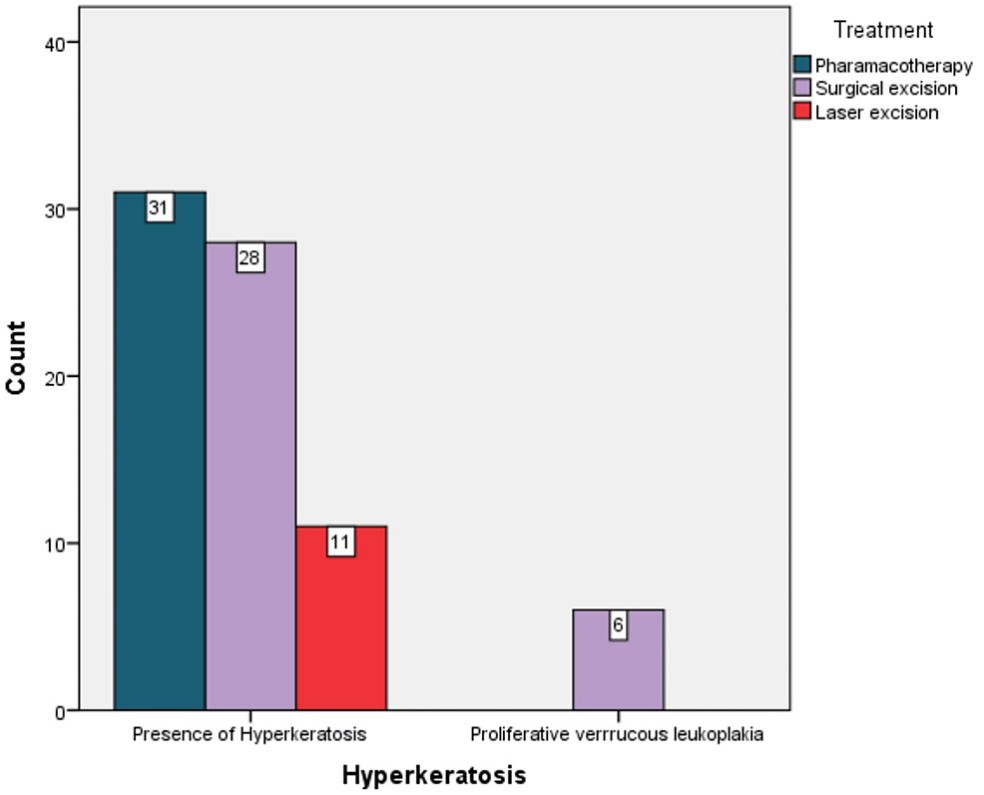
Distribution of treatment among the observed lesions.

Conservative management was utilized for 31 (44%) patients using pharmacotherapy. Lycopene was given to patients for one month, along with dietary supplementation of vitamin A and B12. In total, 28 (39%) patients had undergone surgical excision of the lesion. Eleven (15%) patients underwent laser excision. All six (100%) cases of proliferative verrucous leukoplakia underwent surgical excision. There was a statistically significant difference among patients undergoing treatment between leukoplakia and proliferative verrucous leukoplakia (p = 0.018) (Figure 11).

## Discussion

Leukoplakias are typically asymptomatic and are often detected by healthcare providers during routine examinations. Diagnosis relies on the clinical appearance and exclusion of other oral white lesions. A biopsy examination is essential to rule out specific conditions before confirming a white patch as leukoplakia. Microscopic evidence of dysplasia is not always necessary for diagnosing leukoplakia. However, if a biopsy reveals malignancy or carcinoma in situ, the clinical diagnosis of leukoplakia is no longer applicable, and the histological diagnosis of malignancy takes precedence [9]. Based on the results obtained from the present study, oral leukoplakia was commonly seen in patients between the ages of 41 and 60 years. Male patients were more commonly associated with oral leukoplakia when compared to female patients. However, proliferative verrucous leukoplakia was exclusively observed only in female patients. Patients who had presented with oral leukoplakia had a predisposing etiological factor such as smoking and usage of smokeless tobacco such as betel nut, paan, hans, etc. A large number of patients had a history of tobacco usage for more than 10 years, while few claim to have quit the habit for a couple of years before the diagnosis. Alcohol consumption was also noted in a small set of patients. The most common site of involvement was on the right buccal mucosa, followed by the left buccal mucosa, and bilateral involvement of both the right and left buccal mucosa. A small percentage of cases were found to arise from lip and palate. Predominantly, most patients presented with a homogeneous non-scrapable white plaque or patch, few patients had non-homogeneous speckled plaque or patch, and verrucous and nodular growth were the least commonly observed and only evident in the study subjects. Features of epithelial dysplasia were commonly associated with oral leukoplakia, of which moderate epithelial dysplasia was more commonly observed, followed by mild and severe dysplasia. A small percentage of the included study subjects were diagnosed with proliferative verrucous leukoplakia, and all patients who were diagnosed with proliferative verrucous leukoplakia, progressed to oral squamous cell carcinoma. Among the patients with oral leukoplakia in the present study, two patients had oral leukoplakia with superadded candidal infection and were treated accordingly.

Despite numerous evidence available for the treatment of oral leukoplakia, each patient requires a tailor-made treatment that caters to their needs and requirements of the hour. Predominantly, patients with oral leukoplakia were managed conservatively with pharmacotherapy. Patients were given medications containing lycopene along with antioxidants and vitamin supplements. Patients were also consulted for anti-tobacco counseling and were advised to quit adverse habits such as tobacco consumption both in the form of smoking or smokeless forms as well as alcohol consumption if present. Patients were also advised to avoid spicy foods to control and alleviate the burning sensation. In patients with oral submucous fibrosis, physiotherapy such as balloon blowing and oil pulling was also given. Additionally, in patients with oral submucous fibrosis, intralesional injection of hyaluronidase and dexamethasone was administered. In cases where the pharmacotherapy did not produce adequate mouth opening and relief for the patients, bilateral fiberotomy was done and closed using the buccal pad of fat. A substantial set of patients underwent surgical excision of the lesion either by conventional means or using lasers in a few cases.

In a previous study by Petti et al. [10], the global incidence was estimated to be between 1.7% and 2.7%. The incidence of leukoplakia in India ranged from 0.21% to 5.22%. The prevalence ranged between 3.1% and 3.4% in the three years among our studied population. In this study, males were commonly affected with oral leukoplakia when compared to females, and patients aged 41 to 60 years had a higher incidence of leukoplakia, which was similar to the findings of Venkat et al. [11]. The most common site of involvement of oral leukoplakia was the buccal mucosa in previous studies by Ramya et al. [12], Sivakumar et al. [13], and Venkat et al. [11], followed by the tongue and lip, which was concordant with the present study. This could be attributed to the presence of a high number of patients with smokeless tobacco habits.

In this study, homogenous white patch/plaque type of oral leukoplakia was commonly observed when compared to non-homogeneous speckled variant, proliferative verrucous leukoplakia, and nodular types of leukoplakia were observed in rare instances. Proliferative verrucous leukoplakia was only observed in females in the present study. Proliferative verrucous leukoplakia was first described by Hansen in 1985, and it is an aggressive form of idiopathic leukoplakia that possesses a higher potential for malignant transformation [14]. The malignant transformation potential of proliferative verrucous leukoplakia is 87% to 100% and poses a high mortality rate. It has been hypothesized by Kresty et al. that the aggressive nature of proliferative verrucous leukoplakia can be due to the increased alterations of INK4a/ARF locus as well as increases in p16INK4a and p14ARF homozygous deletion rates [15]. Proliferative verrucous leukoplakia has a higher tendency to occur in elderly females following the results of this study.

In this study, predominantly, leukoplakia was associated with dysplastic features, commonly moderate dysplasia was found to occur in high incidence with leukoplakia in our study, which was following the previous study by Gopinath et al. [16], wherein dysplastic features were observed in their cohort of cases with oral leukoplakia and the prevalence of carcinomatous foci in cases of leukoplakia was found to be 11.9%. In a study by Ramalingam et al. [17], hyperkeratosis with varying degrees of dysplasia was observed and found to be commonly associated with tobacco usage. Shetty et al. [18] found that the severity of the lesion was directly proportional to the higher bidi index of the patient. High-profile studies by previous researchers showed similar results wherein dysplastic features were commonly associated with leukoplakia. However, the grading of dysplastic features is often subjective and can vary between pathologists.

Tobacco induced pre malignancies are leukoplakia, erythroplakia, oral submucous fibrosis, lichen planus, and palatal changes such as palatal keratosis, ulceration, and pigmentation among reverse smokers, of which leukoplakia and oral submucous fibrosis are highly prevalent [19]. Hallikeri et al. [20], and Kumar et al. [21] found that oral submucous fibrosis and leukoplakia were the most prevalent pre-malignant lesions among the study population. In our study, leukoplakia was primarily assessed and was commonly associated with oral submucous fibrosis. This could be attributed to the high incidences of consumption of smokeless tobacco products. Avinash et al. [22] found that betel quid habit was associated with an increased incidence of oral submucous fibrosis, along with a generalized incidence of leukoplakia in patients with tobacco consumption. In a systematic review by Kumbhalwar et al. [23], the pooled prevalence of leukoplakia and oral submucous fibrosis was 6.7% and 4.5%, respectively.

In this study, 6.4% of the observed cases progressed to squamous cell carcinoma, and the malignant transformation was observed in cases of proliferative verrucous leukoplakia. In the systematic review by Pinto et al. [24], it was observed that female patients had a high chance of undergoing malignant transformation, which was following the results obtained by the present study. In a study by Mondal et al. [25], higher Ki-67 proliferative rates were associated with dysplastic and carcinomatous changes associated with oral leukoplakia patches. Thus, it has been hypothesized that Ki-67 proliferative index rates can be indicative of malignant transformation rates of leukoplakia, aiding early diagnosis, treatment, and strict follow-up of such cases.

For the treatment of oral leukoplakia, numerous non-surgical treatments have been employed, predominantly lycopene has been commonly used, followed by retinoids, carotenoids, as well as photodynamic therapy [26]. Lycopene is a carotenoid that lacks provitamin A action, and previous studies have demonstrated a reduced risk of development of degenerative diseases during the consumption of lycopene. Lycopene has high antioxidant properties which target free radicals and reactive oxygen species. Previous studies in an in vitro setting suggested that human neoplastic cellular growth has been inhibited by the usage of lycopene [27]. Surgical excision remains one of the most common treatment modalities for oral leukoplakia and includes conventional surgery, cryosurgery, laser excision, and electrocoagulation [6]. This was concordant with the results of the present study and surgical excision was predominantly performed for patients with oral leukoplakia.

The primary goal of leukoplakia treatment is to avert the lesion’s malignant transformation and restore patient function. There is no universally preferred first line of treatment for leukoplakia as each lesion requires a tailored approach. Besides surgical excision, pharmacotherapy, and laser excision, a new treatment option has recently emerged, i.e., topical 5-aminolevulinic acid-mediated photodynamic therapy, offering a fourth alternative for managing leukoplakia [28]. Healthcare providers, especially dentists, should actively assess the tobacco usage habits of patients and participate in tobacco prevention measures, especially during follow-up periods, as tobacco habits, especially the usage of smokeless tobacco habits, have higher rates of malignant changes [29]. Artificial intelligence can play a crucial role in estimating and predicting treatment outcomes [30] and there is a pressing need for novel systemic anti-cancer medication for effective management [31].

The limitations of the present retrospective study could be that the patients were included only from a single institution and may not be a representative sample of the Indian population. Most patients in this study were lost to follow-up. Cases of proliferative leukoplakia were only a small subset of the included cases and detailed analysis could not be performed. Proliferation markers such as immunohistochemical evaluation of Ki-67 or serological tests were not included for comparison. We also did not compare the treatment outcomes of various options.

## Conclusions

Within the limitations of the present study, leukoplakia was commonly associated with features of dysplasia and oral submucous fibrosis. Leukoplakia was frequently associated with moderate epithelial dysplasia in our study. A small subset of patients also had oral submucous fibrosis and oral leukoplakia with varying grades of dysplasia. Patients were predominantly treated using surgical excision. Patients were treated using lycopene for one month wherein the supplementation was given along with other vitamin supplements.
